# Association between the *PINX1* and *NAT2* polymorphisms and serum lipid levels

**DOI:** 10.18632/oncotarget.23123

**Published:** 2017-12-09

**Authors:** Qing-Hui Zhang, Rui-Xing Yin, Feng Huang, De-Zhai Yang, Wei-Xiong Lin, Shang-Ling Pan

**Affiliations:** ^1^ Department of Cardiology, Institute of Cardiovascular Diseases, The First Affiliated Hospital, Guangxi Medical University, Nanning 530021, Guangxi, People’s Republic of China; ^2^ Department of Molecular Genetics, Medical Scientific Research Center, Guangxi Medical University, Nanning 530021, Guangxi, People’s Republic of China; ^3^ Department of Pathophysiology, School of Premedical Sciences, Guangxi Medical University, Nanning 530021, Guangxi, People’s Republic of China

**Keywords:** the PIN2/TERF1-interacting telomerase inhibitor 1, the N-acetyltransferase 2, single nucleotide polymorphism, lipids, environmental factor

## Abstract

Jing nationality is a relatively conservative and isolated minority in China. Little is known about the association of the PIN2/TERF1-interacting telomerase inhibitor 1 (*PINX1*) and N-acetyltransferase 2 (*NAT2*) single nucleotide polymorphisms (SNPs) and serum lipid levels in the Chinese populations. This study aimed to clarify the association of 6 SNPs of the *PINX1* and *NAT2* and serum lipid levels in two Chinese populations. Genotyping of the SNPs was performed in 1236 Han subjects and 1248 Jing participants. Allelic and genotypic frequencies of these variants (except *NAT2* rs1799931) were different between the two ethnic groups. The minor allele carriers had higher triglyceride (TG, rs11776767, rs1495743 and rs1799930), low-density lipoprotein cholesterol (rs6601530) levels and the apolipoprotein (Apo)A1/ApoB ratio (rs1495743) in Han nationality; and higher total cholesterol (rs1961456), TG (rs11776767, rs6601530 and rs1495743) and lower ApoA1 (rs6601530 and rs1799931) levels in Jing minority than the minor allele non-carriers. The SNPs were not statistically independent by the multiple-locus linkage disequilibrium analyses. The integrative haplotypes and gene-by-gene (G × G) interactions on serum lipid traits were also observed in the two populations. Association analysis based on haplotypes and G × G interactions might be powerful than single-locus tests. Differences in serum lipid profiles between the two populations might partially be attributed to these SNPs, their haplotypes and G × G interactions.

## INTRODUCTION

Epidemiological studies show that cardiovascular disease (CVD) remains the major cause of death globally [1]. Concerted effort is being taken to reduce this disease burden, especially in developed nations [2, 3]. As an important CVD risk factor and target for therapeutic intervention [4], dyslipidemia is considered as a complex disease caused by multiple environmental factors, including age, gender, smoking, drinking and diabetes, and genetic factors [5–7]. Thus, the discovery of the mutations regulating the serum lipid profiles is very vital in the development of new markers for risk evaluation, diagnosis, and prognosis prediction of CVD.

Recently, genome-wide association studies (GWASes) have found compelling genes for modifying lipid metabolism, including the PIN2/TERF1 interacting, telomerase inhibitor 1 (*PINX1*, Gene ID: 54984, OMIM: 606505, formerly known as *LPTL* and LPTS) and the N-acetyltransferase 2 (*NAT2*, Gene ID: 10, OMIM: 612182, formerly known as *AAC2*, *PNAT* and *NAT-2*). The two genes are mapped to the long arm of chromosome 8 closely. *PINX1* is a multifunctional gene at human chromosome 8p23, a region frequently correlated to loss of heterozygosity in various human malignancies [8–10]. It has been confirmed that *PINX1* deficiency could lead to telomerase activation, telomere elongation and chromosome instability [11], however overexpression of *PINX1* caused a decrease in both telomerase activity and cancer cell tumorigenicity [12–14]. *NAT2* is located on chromosome 8p22 and is polymorphous enzymes with vital roles in the deactivation or activation of multitudinous xenobiotics in humans [15–17]. Owing to expression of the isoenzyme in the liver, the genetic mutations of *NAT2* have principally effect on drug metabolism, response and toxicity. *NAT2* genotype has a slow, intermediate or rapid acetylation phenotype, leading to differences in drug metabolic rates and susceptibility to drug toxicity [18–22]. Many GWASes and target single nucleotide polymorphisms (tag SNPs) studies have found significant polymorphisms of these two genes in different ethnic groups. The most frequent mutations, rs11776767 for *PINX1*, rs1961456 and rs1495743 for *NAT2* were associated with blood lipid concentration in many nationalities and the directions of effect were diverse among the different ethnic groups [23–26]. However, whether the association of *PINX1* and *NAT2* and serum lipid variables in the populations of China or whether it shows ethnic-specificity still needs to be explored.

As a multi-nationality country, China has 56 nationalities. Han is the main Chinese nationality distributed all over the country and Jing is one of the 55 minorities living in the Guangxi Zhuang Autonomous Region of south China with a very small size of population. Jing can be traced back to the early 16th century when their ancestors immigrated to China from Vietnam. Almost all of the Jing population now reside in the three islands of Wutou, Shanxin and Wanwei in Jiangping Town, Dongxing City [27]. Jing is the only coastal fishing minority in China and is a relatively isolated ethnic group. Their culture of endogamy is still preserved, which indicates that there are many differences in dietary habits and social customs between Jing and Han (and the other inland nationalities). Genetic heterogeneity may be less within the population. Previous studies have demonstrated that mutations in several lipid-related genes had different association with serum lipid traits between the Jing and Han nationalities and their sex subgroups [28, 29]. Therefore, using the method of tag SNPs combined with recent research reports, we selected these SNPs in the *PINX1* (rs11776767 and rs6601530) and *NAT2* (rs1961456, rs1495743, rs1799930 and rs1799931) to clarify the association of them and environmental factors with serum lipid profiles in the Jing and Han ethnic groups. In addition, we also wanted to explore whether the association analysis of the SNPs based on haplotypes and G × G inter-locus interactions might be powerful than single-locus tests.

## RESULTS

### Subject characteristics

The levels of weight, waist circumference, body mass index (BMI), total cholesterol (TC) and triglyceride (TG) were higher, whereas the values of Apolipoprotein (Apo) A1, the ApoA1/ApoB ratio, diastolic blood pressure and the percentage of individuals who drank alcohol were lower in Jing than in Han populations (*P* < 0.05–0.001; Table 1). No significant difference was observed between the two ethnic groups in serum high-density lipoprotein cholesterol (HDL-C), low-density lipoprotein cholesterol (LDL-C) and ApoB levels (*P* > 0.05 for all).

**Table 1:** Comparison of demographic, lifestyle characteristics and serum lipid levels between the Han and Jing populations

Parameter	Han	Jing	*t* (*x*^2^)	*P*
Number	1236	1248	-	-
Male/female	603/633	614/634	0.042	0.837
Age (years)^1^	58.52±12.97	57.92±13.76	1.116	0.265
Height (cm)	157.05±7.93	157.65±7.84	-1.877	0.061
Weight (kg)	56.16±9.40	58.47±10.00	-5.922	3.63E-9
Body mass index (kg/m^2^)	22.73±3.18	23.46±3.22	-5.700	1.34 E-8
Waist circumference (cm)	77.44±8.82	80.12±9.32	-7.357	2.54 E-13
Smoking status [*n* (%)]				
Non-smoker	1008(81.6)	1039(83.3)		
≤ 20 cigarettes/day	59(4.8)	53(4.2)		
> 20 cigarettes/day	169(13.7)	156(12.5)	1.25	0.534
Alcohol consumption [*n* (%)]				
Non-drinker	993(80.3)	1062(85.1)		
≤ 25 g/day	59(4.8)	99(7.9)		
> 25 g/day	184 (14.9)	87(7.0)	47.106	5.90E-11
Systolic blood pressure (mmHg)	132.77±19.25	131.33±21.60	1.749	0.080
Diastolic blood pressure (mmHg)	81.48±10.26	80.62±10.72	2.050	0.040
Pulse pressure (mmHg)	51.29±15.57	50.71±17.16	0.871	0.384
Glucose (mmol/L)	6.72±1.14	6.76±1.78	-0.703	0.482
Total cholesterol (mmol/L)	4.96±0.89	5.19±0.90	-6.322	3.04E-10
Triglyceride (mmol/L)^2^	1.34(0.65)	1.40(0.76)	-2.565	0.010
HDL-C (mmol/L)	1.79±0.52	1.82±0.46	-2.105	0.136
LDL-C (mmol/L)	2.87±0.44	2.82±0.42	2.630	0.143
ApoA1 (g/L)	1.33±0.20	1.30±0.24	1.917	0.002
ApoB (g/L)	1.04±0.24	1.06±0.25	-2.009	0.132
ApoA1/ApoB	1.34±0.37	1.29±0.38	2.759	0.003

*HDL-C*, high-density lipoprotein cholesterol; *LDL-C*, low-density lipoprotein cholesterol; *Apo*, apolipoprotein. ^1^Mean ± SD determined by *t*-test. ^2^Because of not normally distributed, the value of triglyceride was presented as median (interquartile range), the difference between the two ethnic groups was determined by the Wilcoxon-Mann-Whitney test.

### Genotyping

The polymerase chain reaction (PCR) products of *PINX1* rs11776767, *PINX1* rs6601530, *NAT2* rs1961456, *NAT2* rs1495743, *NAT2* rs1799930 and *NAT2* rs1799931 SNPs were 396-, 351-, 164-, 537-, 328- and 429-bp nucleotide sequences after electrophoresis; respectively (Supplementary Figure 1). Using restriction fragment length polymorphism (RFLP) reaction and then analyzed by agarose gel electrophoresis, according to the presence or absence of the enzyme restriction sites, the genotypes of these SNPs were detected (Supplementary Figure 2).

### Sequencing

The nucleotide direct sequencing confirmed the genotypes shown by PCR-RFLP; respectively (Supplementary Figure 3).

### Genotype and allele distribution

Significant differences were observed between the two ethnic groups in the genotypic and allelic frequencies of the SNPs (*P* < 0.05–0.001; Tables 2 and 3). All variations exhibited the Hardy-Weinberg equilibrium (HWE; *P* > 0.05 for all).

**Table 2:** Genotype frequencies of 6 *PINX1* and *NAT2* SNPs between the Han and Jing ethnic groups [n (%)]

SNP	Genetype	Han (n=1236)	Jing (n=1248)	*χ*^2^	*P*
*PINX1* rs11776767 G>C	GG	749 (60.6)	693 (55.5)		
	GC	413(33.4)	474(38.0)		
	CC	74(6.0)	81(6.5)	6.628	0.036
	*P*_HWE_	0.094	0.657		
*PINX1* rs6601530 G>A	GG	485(39.2)	425(34.0)		
	GA	563(45.6)	598(48.0)		
	AA	188(15.2)	225(18.0)	8.268	0.016
	*P*_HWE_	0.244	0.562		
*NAT2* rs1961456 G>A	GG	667(54.0)	597(47.8)		
	GA	468(37.9)	546(43.7)		
	AA	101(8.1)	105(8.4)	8.897	0.007
	*P*_HWE_	0.142	0.203		
*NAT2* rs1495743 G>C	GG	398(32.2)	472(38.2)		
	GC	633(51.2)	591(47.8)		
	CC	205(16.6)	185(15.0)	11.403	0.003
	*P*_HWE_	0.080	0.232		
*NAT2* rs1799930 G>A	GG	753(60.9)	696(55.8)		
	GA	427(34.5)	468(37.5)		
	AA	56(4.5)	84(6.7)	9.663	0.009
	*P*_HWE_	0.645	0.658		
*NAT2* rs1799931 G>A	GG	957(77.4)	994(78.4)		
	GA	263(21.3)	241(19.6)		
	AA	16(1.3)	13(1.9)	1.914	0.384
	*P*_HWE_	0.664	0.704		

*PINX1*, PIN2/TERF1-interacting telomerase inhibitor 1; *NAT2*, N-acetyltransferase 2; *HWE*, Hardy-Weinberg equilibrium.

**Table 3:** Allele frequencies of 6 *PINX1* and *NAT2* SNPs between the Han and Jing populations [n (%)]

SNP	Allele	Han(n=1236)	Jing(n=1248)	*χ*^2^	*P*
*PINX1* rs11776767	G/C	1911(77.3)/561(22.7)	1860(74.5)/636(25.5)	5.273	0.022
*PINX1* rs6601530	G/A	1533(62.0)/939(38.0)	1448(58.0)/1048(42.0)	8.287	0.004
*NAT2* rs1961456	G/A	1802(72.9)/670(27.1)	1740(69.7)/756(30.3)	6.156	0.013
*NAT2* rs1495743	G/C	1429(57.8)/1043(42.2)	1535(61.5)/961(38.5)	7.030	0.008
*NAT2* rs1799930	G/A	1933(78.2)/539(21.8)	1860(75.2)/636(24.8)	9.279	0.002
*NAT2* rs1799931	G/A	2177(88.1)/295(11.1)	2229(89.3)/267(10.7)	1.893	0.169

*PINX1*, PIN2/TERF1-interacting telomerase inhibitor 1; *NAT2*, N-acetyltransferase 2.

### Single SNP and serum lipid profiles

**Figure 1:**
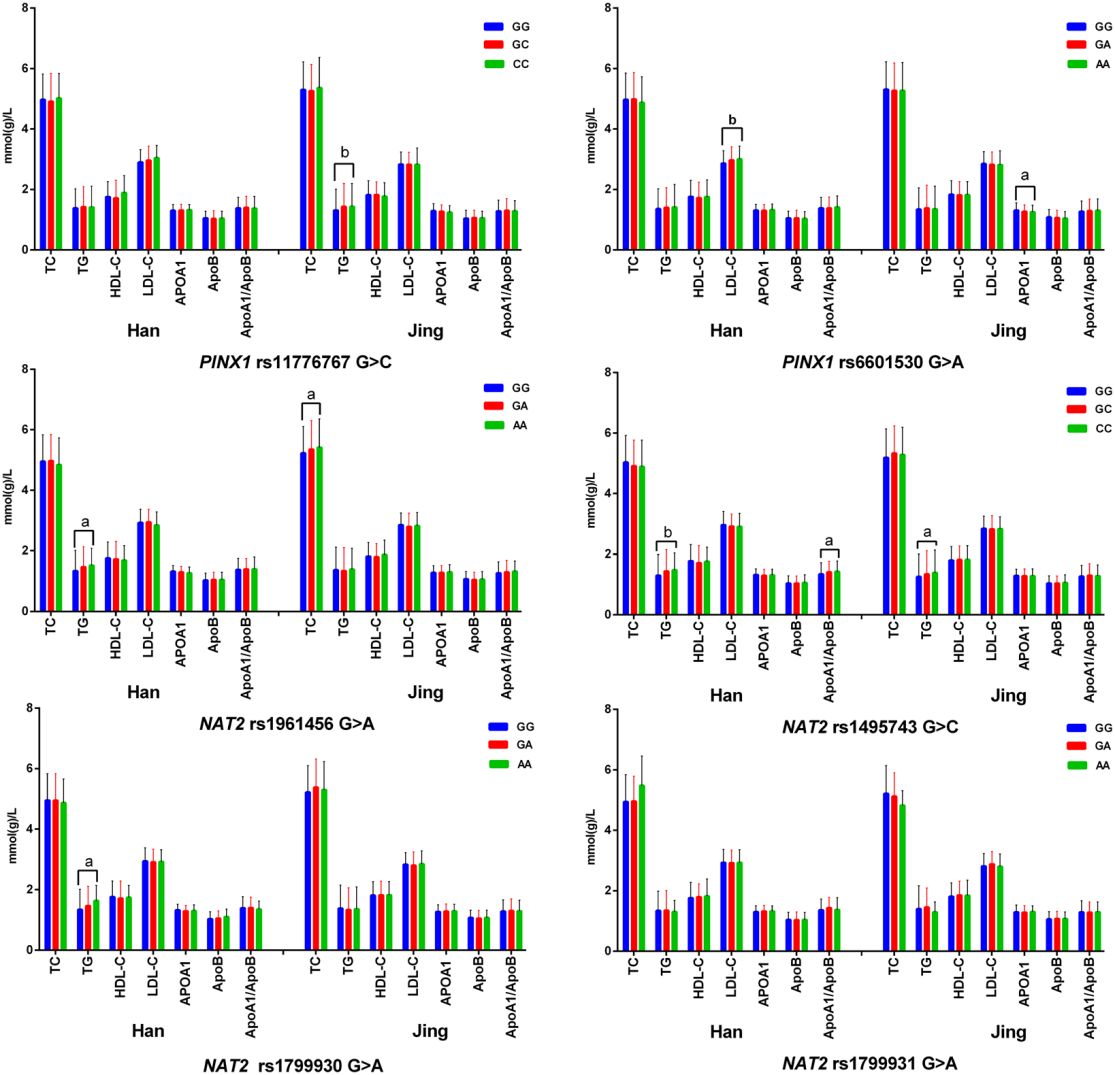
Association of single SNP and serum lipid levels *TC*, total cholesterol; *TG*, triglyceride; *HDL-C*, high-density lipoprotein cholesterol; *LDL-C*, low-density lipoprotein cholesterol; *Apo*, apolipoprotein. ^a^*P* < 0.008 (corresponding to *P* < 0.05 after adjusting for 6 independent tests by the Bonferroni correction, this value was considered statistically significant) and ^b^*P* < 0.001.

As shown in Figure 1, the minor allele carriers had higher the levels of TG (rs11776767, rs1495743, and rs1799930), LDL-C (rs6601530) and the ratio of ApoA1 to ApoB (rs1495743) in Han nationality; and higher TC (rs1961456), TG (rs11776767, rs6601530 and rs1495743), and lower ApoA1 (rs6601530 and rs1799931) levels in Jing minority than the minor allele non-carriers (*P* < 0.008 for all).

### Haplotypes and serum lipid profiles

**Figure 2:**
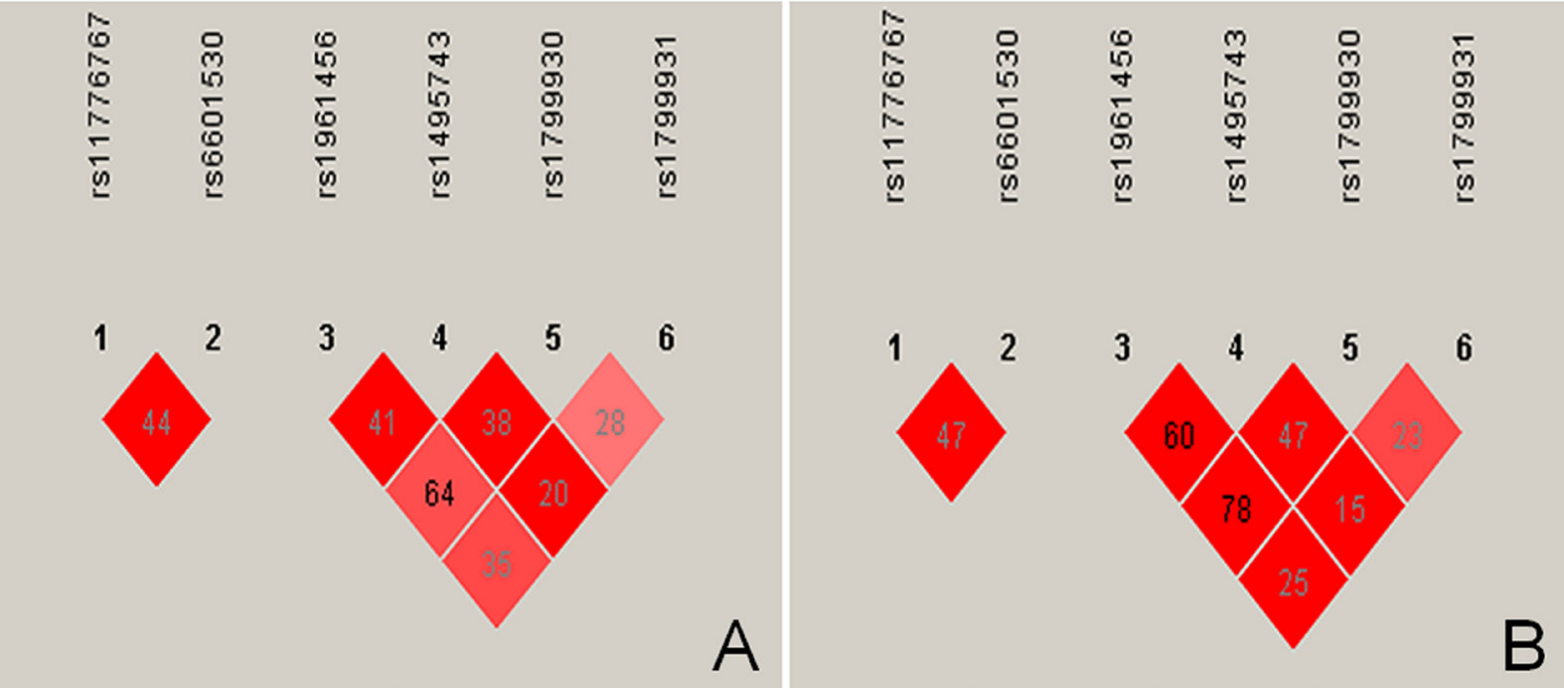
Linkage disequilibrium (LD) analyses of the *PINX1* and *NAT2* SNPs LD among the (1) *PINX1* rs11776767, (2) *PINX1* rs6601530, (3) *NAT2* rs1961456, (4) *NAT2* rs1495743, (5) *NAT2* rs1799930, (6) *NAT2* rs1799931SNPs in the Han **(A)**, Jing **(B)**. The LD status is expounded by the *r*^2^.

**Figure 3:**
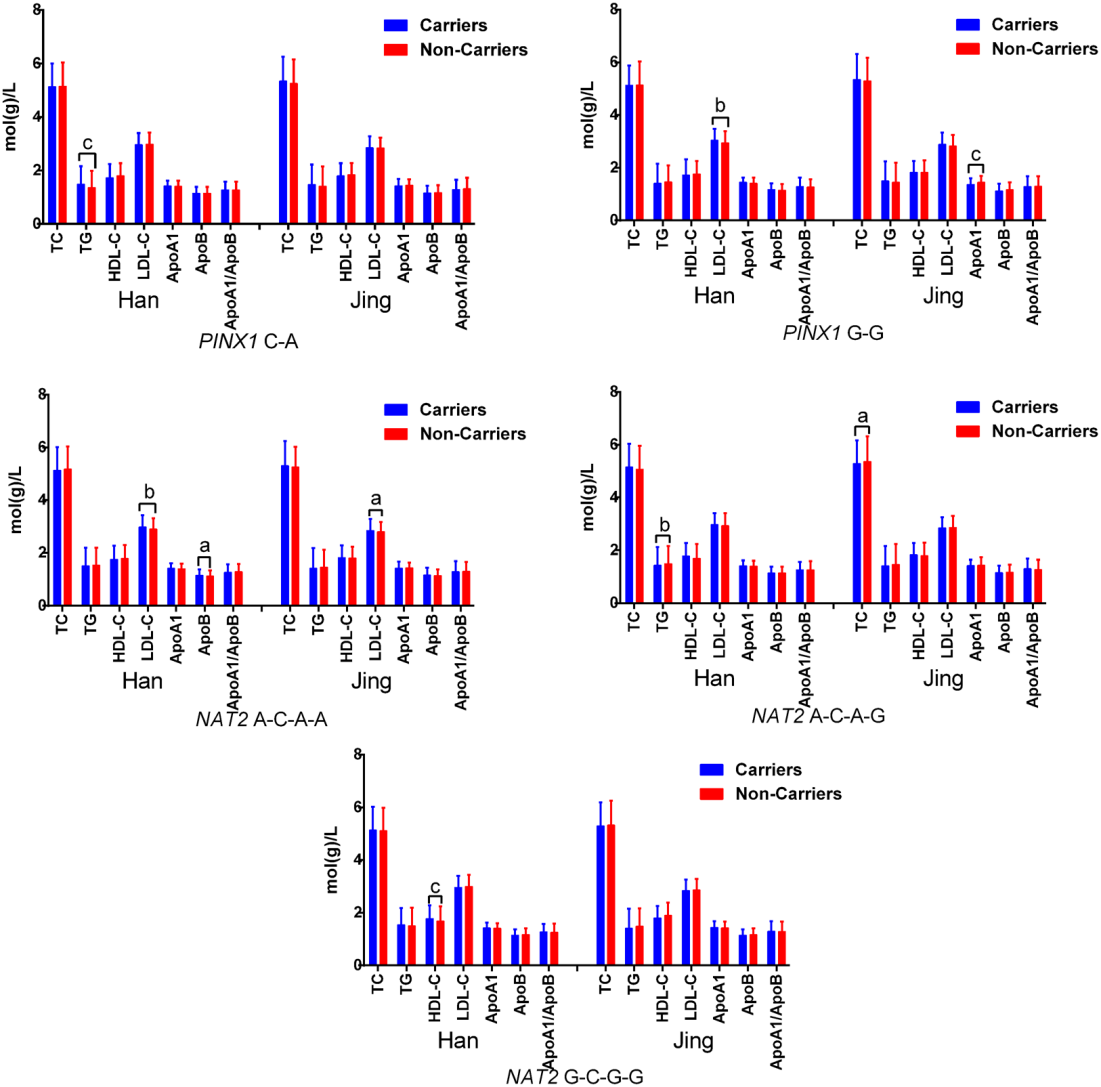
Association of the haplotypes and serum lipid traits The haplotype was presented as *PINX1* rs11776767-rs6601530 and *NAT2* rs1961456-rs1495743-rs1799930-rs1799931. *TC*, total cholesterol; *TG*, triglyceride; *HDL-C*, high-density lipoprotein cholesterol; *LDL-C*, low-density lipoprotein cholesterol; *Apo*, apolipoprotein. ^a^*P* < 0.05, ^b^*P* < 0.01 and ^c^*P* < 0.001.

The tested SNPs were not statistically independent by the multiple-locus linkage disequilibrium (LD) analyses in each population (Figure 2). The commonest haplotypes were *PINX1* G-G and *NAT2* G-G-G-G (> 50% of the individuals; Table 4). Significant differences were observed between the Jing and Han nationalities in the frequencies of the *PINX1* C-A, *PINX1* G-G, *NAT2* A-C-A-A, *NAT2* A-C-A-G and *NAT2* G-C-G-G haplotypes (*P* < 0.05–0.001). As shown in the Figure 3, these haplotypes were associated with serum lipid levels. The correlation algorithm based on haplotypes was more powerful for finding more precise and distinct markers than single-locus tests.

**Table 4:** Haplotype frequencies of 6 *PINX1* and *NAT2* SNPs in the Han and Jing populations [n (frequency)]

Haplotype	Han	Jing	χ^2^	*P*-value	Odd Ratio[95% CI]
*PINX1* C-A	551.99(0.223)	635.98(0.255)	6.775	0.009261	0.841 [0.738∼0.958]
*PINX1* G-A	384.01(0.155)	408.02(0.163)	0.612	0.434140	0.941 [0.808∼1.096]
*PINX1* G-G	1535.99(0.621)	1451.98(0.582)	8.137	0.004350	1.180 [1.053∼1.322]
*NAT2* A-C-A-A	276.00(0.112)	227.97(0.091)	6.504	0.010793	1.272 [1.057∼1.531]
*NAT2* A-C-A-G	288.00(0.117)	408.03(0.163)	20.827	5.15E-6	0.686 [0.583∼0.807]
*NAT2* A-C-G-A	59.98(0.024)	23.99(0.010)	-	-	-
*NAT2* A-C-G-G	72.00(0.029)	96.01(0.038)	2.969	0.084900	0.761 [0.558∼1.039]
*NAT2* G-C-G-G	372.02(0.150)	288.00(0.115)	14.870	0.000116	1.383 [1.172∼1.631]
*NAT2* G-G-G-G	1403.97(0.568)	1451.96(0.582)	0.141	0.707658	0.978 [0.873∼1.096]
*NAT2* G-G-G-A	0.00(0.000)	0.03(0.000)	-	-	-

The haplotype is combined with *PINX1* rs117767667-rs6601530, *NAT2* rs1961456-rs1495743-rs1799930-rs1799931. *PINX1*, PIN2/TERF1-interacting telomerase inhibitor 1; *NAT2*, N-acetyltransferase 2.

### G × G interactions on serum lipid profiles

**Figure 4:**
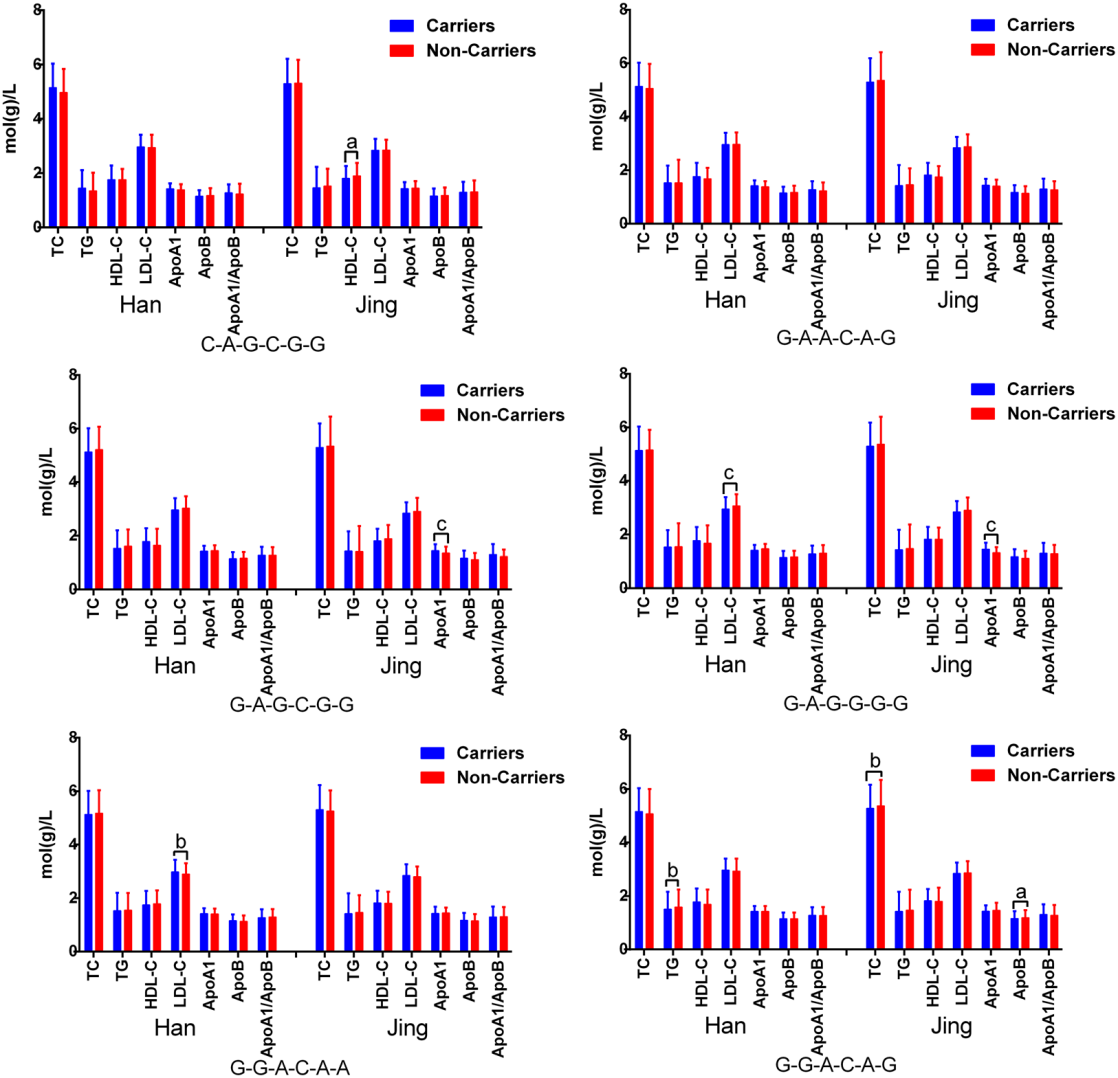
G × G interactions of the *PINX1* and *NAT2* SNPs on serum lipid levels The order was presented as rs11776767-rs6601530-rs1961456-rs1495743-rs1799930-rs1799931. ^a^*P* < 0.05, ^b^*P* < 0.01 and ^c^*P* < 0.001.

The G × G interaction of G-G-G-G-G-G was the commonest one (> 30% of the individuals; Table 5). Significant differences were observed between Jing and Han populations in the frequencies of the C-A-G-G-G-C, G-A-A-C-A-G, G-A-G-C-G-G, G-A-G-G-G-G, G-G-A-C-A-A and G-G-A-C-A-G (*P* < 0.05–0.001). The G × G interactions on blood lipid profiles are illustrated in Figure 4. The association test based on G × G interactions outperformed other more common single variant association approaches.

**Table 5:** G × G interaction frequencies of 6 *PINX1* and *NAT2* SNPs in the Han and Jing populations [n (frequency)]

G × G inteaction						Han	Jing	χ^2^	*P*-value	Odd Ratio[95%CI]
A	B	C	D	E	F					
C	A	G	G	G	C	92.87(0.038)	131.96(0.053)	6.531	0.010621	0.703 [0.535∼0.922]
C	A	G	G	G	G	456.00(0.184)	504.00(0.202)	2.173	0.140400	0.899 [0.780∼1.036]
C	A	G	C	G	G	3.13(0.001)	0.03(0.000)	-	-	-
G	G	A	C	A	A	12.00(0.005)	15.56(0.006)	-	-	-
G	A	A	C	A	G	36.00(0.015)	108.00(0.043)	36.029	2.00E-009	0.328 [0.224∼0.480]
G	A	A	C	G	A	0.00(0.000)	23.98(0.010)	-	-	-
G	A	A	C	G	G	36.00(0.015)	60.01(0.024)	-	-	-
G	A	G	C	G	G	183.13(0.074)	60.05(0.024)	67.690	2.01E-016	3.275 [2.433∼4.410]
G	A	G	G	G	G	96.00(0.039)	140.42(0.056)	8.095	0.004453	0.681 [0.522∼0.889]
G	A	A	C	A	A	264.00(0.107)	212.40(0.085)	7.121	0.007635	1.295 [1.071∼1.567]
G	G	A	C	A	G	252.00(0.102)	300.04(0.120)	3.950	0.046902	0.835[0.699∼0.998]
G	G	A	C	G	G	36.00(0.015)	36.00(0.014)	-	-	-
G	G	G	C	G	G	116.87(0.047)	95.96(0.038)	2.497	0.114075	1.249 [0.947∼1.646]
G	G	G	G	G	A	0.00(0.000)	0.04(0.000)	-	-	-
G	G	G	G	G	G	828.00(0.335)	807.52(0.324)	1.015	0.313789	1.064 [0.943∼1.199]
G	G	A	G	G	A	24.00(0.010)	0.00(0.000)	-	-	-
G	A	A	C	G	A	36.00(0.015)	0.00(0.000)	-	-	-

*A, PINX1* rs11776767 G>C; *B, PINX1* rs6601530 G>A; *C, NAT2* rs1961456 G>A; *D, NAT2* rs1495743 G>C; *E, NAT2* rs1799930 G>A; *F, NAT2* rs1799931 G>A; *PINX1*, PIN2/TERF1-interacting telomerase inhibitor 1; *NAT2*, N-acetyltransferase 2.

### Integrative SNPs, haplotypes and G × G interactions on serum lipid profiles

The integrative association analysis of the SNPs, haplotypes and G × G interactions on serum lipid profiles in the two populations is described in the Table 6. After the confounding factors, including age, sex, waist circumference, BMI, smoking, drinking, blood pressure and blood glucose, were adjusted, generalized linear models indicated that the SNPs, haplotypes and G × G interactions were significantly associated with serum lipid levels (*P* < 0.05–0.001). In addition, the correlation analysis based on haplotypes and G × G interactions might be more powerful than single-locus tests.

**Table 6:** Association of integrative *PINX1* and *NAT2* mutations, haplotypes and G × G interactions with serum lipid traits in the Han and Jing populations

Lipid	Mutation/Haplotype/G × G interaction	Affected phenotype/ Other phenotype	Unstandardized Coefficients		Standardized Coefficients	*t*	*P*
			Beta	Std.error	Beta		
Han							
TG	*PINX1* rs11776767	G/C	-0.255	0.057	-0.143	-4.507	7.20E-6
	*NAT2* rs1799931	G/A	-0.131	0.065	-0.064	-2.024	0.043
	*PINX1* C-A	Carriers/Non-carriers	-0.154	0.051	-0.086	-3.004	0.003
	G-A-G-G-G-G	Carriers/Non-carriers	0.199	0.085	0.068	2.349	0.019
HDL-C	*PINX1* rs11776767	GG/GC/CC	0.108	0.027	0.124	4.015	6.31E-5
	*NAT2* rs1799931	GG/GA/AA	0.125	0.031	0.123	3.989	7.04E-5
	*NAT2* A-C -A-G	Carriers/Non-carriers	-0.108	0.037	-0.085	-2.946	0.003
	G-A-G-C-G-G	Carriers/Non-carriers	-0.165	0.044	-0.107	-3.717	2.11E-4
LDL	*PINX1* rs6601530	GG/GA/AA	0.069	0.018	0.108	3.819	1.41E-4
	G-G-A-C-A-A	Carriers/Non-carriers	-0.067	0.032	-0.060	-2.085	0.037
	G-A-G-G-G-G	Carriers/Non-carriers	0.107	0.043	0.072	2.520	0.012
ApoA1	*PINX1* G-G	Carriers/Non-carriers	-0.049	0.016	-0.087	-3.047	0.002
	G-A-G-C-G-G	Carriers/Non-carriers	-0.062	0.021	-0.083	-2.906	0.004
ApoB	G-G-A-C-A-A	Carriers/Non-carriers	-0.039	0.016	-0.068	-2.427	0.015
ApoA1/ApoB	*NAT2* rs1495743	GG/GC/CC	-0.072	0.019	-0.110	-3.879	1.11E-4
	*PINX1* C-A	Carriers/Non-carriers	0.062	0.018	0.099	3.402	0.001
	G-A-G-C-G-G	Carriers/Non-carriers	-0.108	0.033	-0.095	-3.244	0.001
Jing							
TC	*G-G-A-C-A-G*	Carriers/Non-carriers	0.154	0.056	0.073	2.736	0.006
TG	*NAT2* rs1495743	G/C	0.166	0.057	0.088	2.936	0.003
	*NAT2* rs1799931	GG/GA/AA	-0.138	0.063	-0.066	-2.186	0.029
	*NAT2* A-C-A-G	Carriers/Non-carriers	0.171	0.054	0.085	3.132	0.002
	*NAT2 G-C-G-G*	Carriers/Non-carriers	0.141	0.066	0.057	2.132	0.033
HDL-C	*NAT2 rs1961456*	GG/GA/AA	-0.085	0.026	-0.123	-3.291	0.001
	rs1495743	G/A	0.081	0.036	0.084	2.245	0.025
	*NAT2* G-C-G-G	Carriers/Non-carriers	0.077	0.034	0.061	2.242	0.025
ApoA1	*PINX1* rs11776767	GG/GC/CC	0.048	0.013	0.125	3.831	1.34E-4
	*PINX1* rs6601530	GG/GA/AA	-0.056	0.011	-0.161	-4.961	7.97E-7
	G-A-G-G-G-G	Carriers/Non-carriers	-0.128	0.021	-0.164	-5.943	3.62E-9
ApoB	*PINX1* rs6601530	GG/GA/AA	-0.034	0.012	-0.086	-2.837	0.005
	*NAT2* rs1799931	G/A	-0.052	0.021	-0.075	-2.457	0.014
	G-A-G-G-G-G	Carriers/Non-carriers	-0.097	0.024	-0.110	-3.972	0.000

*HDL-C*, high density lipoprotein cholesterol; *LDL-C*, low density lipoprotein cholesterol; *Apo*, apolipoprotein; *PINX1*, PIN2/TERF1-interacting telomerase inhibitor 1; *NAT2*, N-acetyltransferase 2.

## DISCUSSION

In this study, the main results are as follows: (i) the genotype and allele distributions of 6 SNPs in the *PINX1* and *NAT2* and the distributions of the haplotypes and the G × G interactions among the SNPs showed significant differences between the Jing and Han nationalities; (ii) the results of the integrative SNPs, haplotypes and G × G interactions indicated that there was probable interaction of the *PINX1* and *NAT2* SNPs on blood lipid levels; and (iii) it proved that association analysis based on haplotypes and G × G interactions might be more powerful than single-locus tests.

As an important predictor of CVD, dyslipidemia is a complex and multifactorial disease induced by multiple environmental factors, including age, sex, obesity, cigarette smoking, alcohol consumption, hypertension, diet and exercise [30, 31] and lots of genetic factors such as lipid-related gene mutations; and their interactions [5, 32]. In this study, we described that the values of TC and TG were higher, while the values of ApoA1 and the ApoA1/ApoB ratio were lower in Jing than in Han nationalities. These results might be owing to the differences in the lipid-related genes and lifestyle between the two ethnic groups. Of the 56 ethnic groups in China, the Han nationality is the largest one and is extensively distributed all around the country. Among the 55 minorities, Jing is the only coastal fishing minority. They reside in a relatively isolated and conservative environment and have similar eating habits. In such a situation, compared with the other inland ethnic groups, it has very unique cultural characteristics and eating customs. Endogamy is still preserved in Jing and intermarriage with people of Han or other nationalities rarely occurred. Due to its relatively closed system of endogamy and unique customs, we inferred that some inheritance features in Jing possibly showed differences from those in Han. In this study, significant differences were observed between the two populations in the genotype and allele distributions of the *PINX1* rs11776767, *PINX1* rs6601530, *NAT2* rs1961456, *NAT2* rs1495743 and *NAT2* rs1799930 SNPs. All of the detected mutations were in the HWE. In addition, the frequencies of the *PINX1* C-A, *PINX1* G-G, *NAT2* A-C-A-A, *NAT2* A-C-A-G and *NAT2* G-C-G-G haplotypes and the C-A-G-G-G-C, G-A-A-C-A-G, G-A-G-C-G-G, G-A-G-G-G-G, G-G-A-C-A-A and G-G-A-C-A-G G × G interactions also showed quantitative significantly differences between the two populations. These findings indicated that the distributions of the detected 6 SNPs of *PINX1* and *NAT2*, their haplotypes and G × G interactions possibly had a racial/ethnic-specificity and these genetic heterogeneities might be correlated to the differences between the Jing and Han nationalities in serum lipid profiles.

The locations of the *PINX1* and *NAT2* are very close, both of which are on the chromosome 8p. Data have showed that *PINX1* has functional role in inhibition of telomerase activity and reduction of telomere length [33]. The expression of *PINX1* significantly decreased some human cancers and was correlated to the adverse outcome of cancer patients. A relevant study [34] showed that the *PINX1* mutations increased the risk of carotid intima media thickness, which is used to determine atherosclerosis, a chronic formation process caused by excessive cholesterol deposition in the arterial intima [35]. Teslovich *et al.* [26] reported the effect of *PINX1* variants on high serum TG concentrations, which was successfully replicated by Willer *et al.* [36]. However, the exact mechanism by which *PINX1* affects serum lipid profiles is still unclear. The protein product of the *NAT2* is capable of N-acetylation and O-acetylation which are implicated in the metabolism and detoxification of naturally occurring xenobiotics, including carcinogens and drugs [37]. The acetylator phenotype is determined by studying the acetylation of variety drugs such as caffeine, dapsone, sulfadimidine or isoniazid. Therefore, the acetylation capacity in humans has been linked to *NAT2* polymorphisms, which alters susceptibility to cancer and other diseases including adverse drug reactions [38, 39]. Al-Shaqha *et al.* reported that the *NAT2* alleles were associated with the type 2 diabetes mellitus [40], which is closely related to the abnormal blood lipid profiles. Studies describing the effect of *NAT2* alleles on serum lipid profiles are few [25, 26] and the specific mechanism of the influence of *NAT2* on serum lipid profiles is still unclear. Because of the close locations of *PINX1* and *NAT2*, they may interact with each other on serum lipid profiles. Therefore, the specific mechanism of *PINX1*, *NAT2* and their interactions on serum lipid profiles still needs to be continued in-depth studied, which probably provide a novel therapeutic target.

The current study indicated that the effect of the *PINX1* and *NAT2* SNPs, their haplotypes and G × G interactions on serum lipid parameters might have a racial/ethnic specificity. Many GWASes and replicated studies have reported that the SNPs near the *PINX1* and *NAT2* were associated with serum lipid phenotypes [23-26, 41]. The *PINX1* rs11776767 SNP has been associated with TG in many nationalities [23, 26], however, the directions of effect were different among different ethnic groups. The minor allele carriers had a positive correlation with TG in East Asian, South Asian and African American, but an opposite effect in European. Likewise, the *NAT2* rs1961456 and *NAT2* rs1495743 SNPs have been associated with TC and TG respectively, and had diverse directions of effect on serum lipid profiles in different populations [26]. In this study, we successfully replicated the association of the *PINX1* rs11776767 and *NAT2* rs1495743 SNPs with TG in both populations; and the *NAT2* rs1961456 SNP with TC in the Jing minority. The minor allele carriers of *PINX1* rs11776767, *NAT2* rs1495743 and rs1961456 SNPs consensually had higher serum lipid parameters than the minor allele non-carriers. What’s more, we also explored the minor allele carriers had higher LDL-C (*PINX1* rs6601530), the ratio of ApoA1 to ApoB (*NAT2* rs1495743) and TG (*NAT2* rs1799930) than the minor allele non-carriers in Han. The minor allele carriers of *PINX1* rs6601530 had higher TG and lower ApoA1 than the minor allele non-carriers in Jing minority. Previous studies proved that the strength of a single marker association analysis might suffer for LD information included in flanking markers was neglected. Haplotypes (which can be regarded as a collection of ordered markers) might increase power over individual, unorganised markers [42]. In addition, blood lipid profiles are influenced by multiple genes and their interactions. In this study, multiple-locus LD analyses showed that the tested loci were not statistically independent in both populations. Therefore, the association tests based on the haplotypes and G × G inter-locus interactions have been conducted. We found that the haplotypes and G × G interactions were also associated with serum lipid levels and the association had a same racial specificity. The inconsistent association among different populations might be attributed to genetic heterogeneity.

In addition, environmental factors such as lifestyles and dietary habits are also closely correlated to serum lipid profiles [30, 43]. Jing is a coastal ethnic minority, which makes a living out of fishing [44] and fish is one of the most popular dishes they eat. Fishes are rich in long-chain n-3 (ω-3) polyunsaturated fatty acids (LCn-3PUFA), which is beneficial to serum lipid profiles. However, previous studies also showed that LCn-3PUFA could effectively increase TC, TG and LDL-C concentrations and reduce HDL-C concentrations [45, 46]. In this study, we also showed that the values of waist circumference, weight and BMI were higher, whereas the rate of alcohol consumption was lower in Jing than in Han. A study conducted in young black and white adults showed that a 10-year weight gain tended to have disadvantageous effect on the levels of TG, LDL-C and HDL-C [47]. Williams *et al.* [48] showed that the rise of waist circumference and BMI increased the risk of hypercholesterolemia during the seven-year follow-up. A moderate alcohol consumption was causally related to the low risk of CVD through mainly increasing serum HDL-C and ApoA1 concentrations [49]. However, excessive intake of alcohol has been proved to lead to hypertriglyceridemia [50], which is an important predictor of CVD.

Several potential limitations could not be neglected in this study. First, compared with lots of previous GWASes, the sample size was comparatively less. Next, several confounding factors, including age, sex, BMI, smoking, drinking and blood pressure might have influence on blood lipid profiles among different genotypes in the two populations, although we have adjusted these factors for the statistical analysis. What’s more, the dietary influence on serum lipid profiles could not been eliminated during the statistical analysis. Last but not the least, in this study, we have only detected the association of mutations in the *PINX1* and *NAT2* with blood lipid levels. there are still lots of other unmeasured lipid metabolism-related genes. Thus, more genes and their interactions remain to be determined.

In summary, the *PINX1* and *NAT2* SNPs, their haplotypes and G × G interactions were associated with serum lipid parameters in the Jing and Han nationalities, but the association was different between the two populations. The association analysis based on haplotypes and G × G interactions might be powerful than single-locus tests. Differences in serum lipid profiles between the two ethnic groups might partially be attributed to the *PINX1* and *NAT2* SNPs.

## MATERIALS AND METHODS

### Study populations

The current study included 1236 (603 males, 48.8% and 633 females, 51.2%) unrelated subjects of Han nationality and 1248 unrelated participants (614 males, 49.2% and 634 females, 50.8%) of Jing minority. They were randomly selected from our previous stratified randomized samples. All participants were rural agricultural (Han) and/or fishery (Jing) workers living in Jiangping Town, Dongxing City, Guangxi Zhuang Autonomous Region, People’s Republic of China. The participants’ age ranged from 15–80 years with a mean age of 58.52 ± 12.97 years in Han and 57.92 ± 13.76 years in Jing, respectively. All participants were essentially healthy and had no evidence of diseases related to atherosclerosis, CVD and diabetes. Any participant had a history of taking medications known to affect serum lipid levels (lipid-lowering drugs such as statins or fibrates, beta blockers, diuretics, or hormones) was excluded before the blood sample was taken. The study design was approved by the Ethics Committee of the First Affiliated Hospital, Guangxi Medical University (No: Lunshen-2011-KY-Guoji-001; Mar. 7, 2011). Informed consent was obtained from all participants.

### Epidemiological survey

The epidemiological survey was carried out using internationally standardized method, following a common protocol. Information on demographics, socioeconomic status, and lifestyle factors was collected with standardized questionnaires. Cigarette smoking status was categorized into groups of cigarettes per day: 0, ≤ 20 and > 20 [51]. Alcohol consumption was categorized into groups of grams of alcohol per day: 0, ≤ 25 and > 25 [52]. Several parameters such as blood pressure, height, weight and waist circumference were measured, while BMI (kg/m^2^) was calculated. Hypertension was defined as a systolic blood pressure of 140 mmHg or greater, and/or a diastolic blood pressure of 90 mmHg or higher, or the use of antihypertensive drugs [53]. A BMI less than 18.5, 18.5 to 24, 24 to 28, and greater than 28 kg/m^2^ was defined as underweight, normal weight, overweight and obesity, respectively [54]. Likewise, waist circumference was categorized into normal (≤ 85 cm for males and ≤ 80 cm for females) and abdominal obesity (> 85 cm for males and > 80 cm for females) subgroups [55].

### Biochemical measurements

A fasting venous blood sample of 5 ml was drawn from the participants. The levels of TC, TG, HDL-C and LDL-C in the samples were determined by enzymatic methods with commercially available kits. Serum ApoA1 and ApoB levels were assessed by the immune-turbid metric immunoassay. Fasting blood glucose was determined by glucose meter. The normal values of serum TC, TG, HDL-C, LDL-C, ApoA1 and ApoB levels, and the ratio of ApoA1 to ApoB in our Clinical Science Experiment Center were 3.10-5.17, 0.56-1.70, 1.16-1.42, 2.70-3.10 mmol/L, 1.20-1.60, 0.80-1.05 g/L, and 1.00-2.50; respectively. The individuals with TC > 5.17 mmol/L and/or TG > 1.70 mmol/L were defined as hyperlipidemic [56].

### SNP selection

We selected 6 SNPs in the *PINX1* and *NAT2* with the following assumption: (i) tag SNPs, which were established by Haploview (Broad Institute of MIT and Harvard, Cambridge, MA, USA, version4.2); (ii) functional mutations (http://snpinfo.niehs.nih.gov/snpinfo/snpfunc.htm) in functional areas of the gene fragment from NCBI dbSNP Build 132 (http://www-ncbi-nlm-nih-gov.ezp-prod1.hul.harvard.edu/SNP/); (iii) a known minor allele frequency (MAF) higher than 1% in European ancestry from the Human Genome Project Database; and (iv) mutations might be associated with the lipid-related traits or cardiometabolic risk in the latest studies.

### Genotyping

DNA was isolated from blood samples using DNA Blood Midi kits (Qiagen, Hilden, Germany) following the protocol recommended by the vendor. Six SNPs were genotyped by PCR-RFLP. The characteristics of each SNP and the details of each primer pair, annealing temperature, length of the PCR products and corresponding restriction enzyme used for genotyping are summarized in Supplementary Tables 1 and 2. The PCR products of the samples (two samples of each genotype) were sequenced with an ABI Prism 3100 (Applied Biosystems, International Equipment Trading Ltd., Vernon Hill, IL, USA) in Shanghai Sangon Biological Engineering Technology & Services Co., Ltd., China.

### Statistical analyses

The statistical analysis was performed with the statistical software SPSS 17.0 (SPSS Inc., Chicago, IL, USA). Quantitative variables were presented as the mean ± SD for those, that are normally distributed, whereas the medians and interquartile ranges for TG, which is not normally distributed. General characteristics between the two ethnic groups were compared by Student’s unpaired *t*-test. The frequencies of the genotypes, alleles, haplotypes and G × G interactions between the two ethnic groups were analyzed by the chi-squared test; and the standard goodness-of-fit verified the test HWE. The Pair-wise LD, the frequencies of haplotypes and G × G interactions among the SNPs were calculated using Haploview (version 4.2; Broad Institute of MIT and Harvard). The association of the genotypes, haplotypes and G × G interactions with lipid phenotypic variations was tested by the analysis of covariance (ANCOVA). Any SNPs associated with serum lipid profiles at a value of *P* < 0.008 (corresponding to *P* < 0.05 after adjusting for 6 independent tests by the Bonferroni correction) were considered statistically significant. Generalized linear models were used to assess the association of the genotypes (common homozygote genotype = 0, heterozygote genotype = 1, rare homozygote genotype = 2), alleles (the minor allele non-carrier = 0, the minor allele carrier = 1), haplotypes (the haplotype non-carrier = 0, the haplotype carrier = 1) and G × G interactions (the G × G interaction non-carrier = 0, the G × G interaction carrier = 1) with serum lipid parameters. The factors of age, gender, BMI, waist circumference, systolic blood pressure, diastolic blood pressure, pulse pressure, cigarette smoking, alcohol consumption and fasting blood glucose levels were adjusted for the statistical analysis. The pattern of pair-wise LD between the selected mutations was measured by *D*′ and *r*^2^ using the Haploview software. Two-sided *P* value of less than 0.05 was considered statistically significant for the remaining parameters.

## SUPPLEMENTARY MATERIALS FIGURES AND TABLES


