# Supplementary material for: Association between the PINX1 and NAT2 polymorphisms and serum lipid levels
Source: Oncotarget. 2017 Dec 9;8(69):114081–94. doi: 10.18632/oncotarget.23123 (PMC5768388; doi:10.18632/oncotarget.23123)
Supplement: Supplementary file 1 [file oncotarget-08-114081-s001.pdf]

Association between the *PINX1* and *NAT2* polymorphisms and serum lipid levels

SUPPLEMENTARY MATERIALS

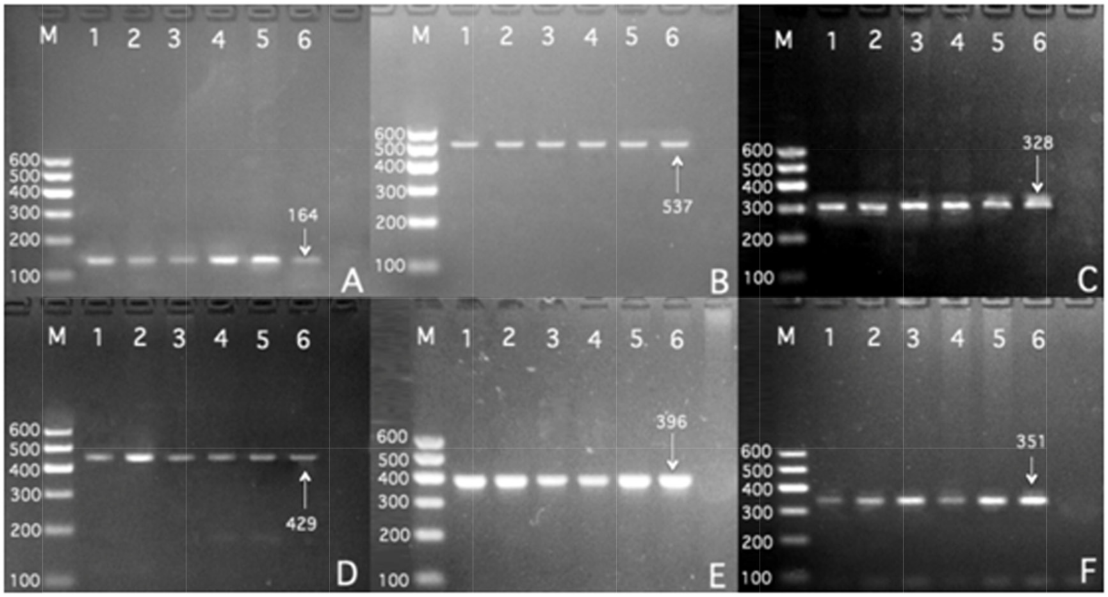

Supplementary Figure 1: Agarose gel electrophoresis (2%) of PCR products of the *PINX1* and *NAT2* SNPs. Lane M: DNA ladder 100 bp; PCR amplicon products of **(A)** *NAT2* rs1961743, **(B)** *NAT2* rs1495743, **(C)** *NAT2* rs1799930, **(D)** *NAT2* rs1799931, **(E)** *PINX1* rs11776767 and **(F)** *PINX1* rs6601530 SNPs were 164-, 537-, 328-, 429-, 396- and 351-bp nucleotide sequences; respectively.

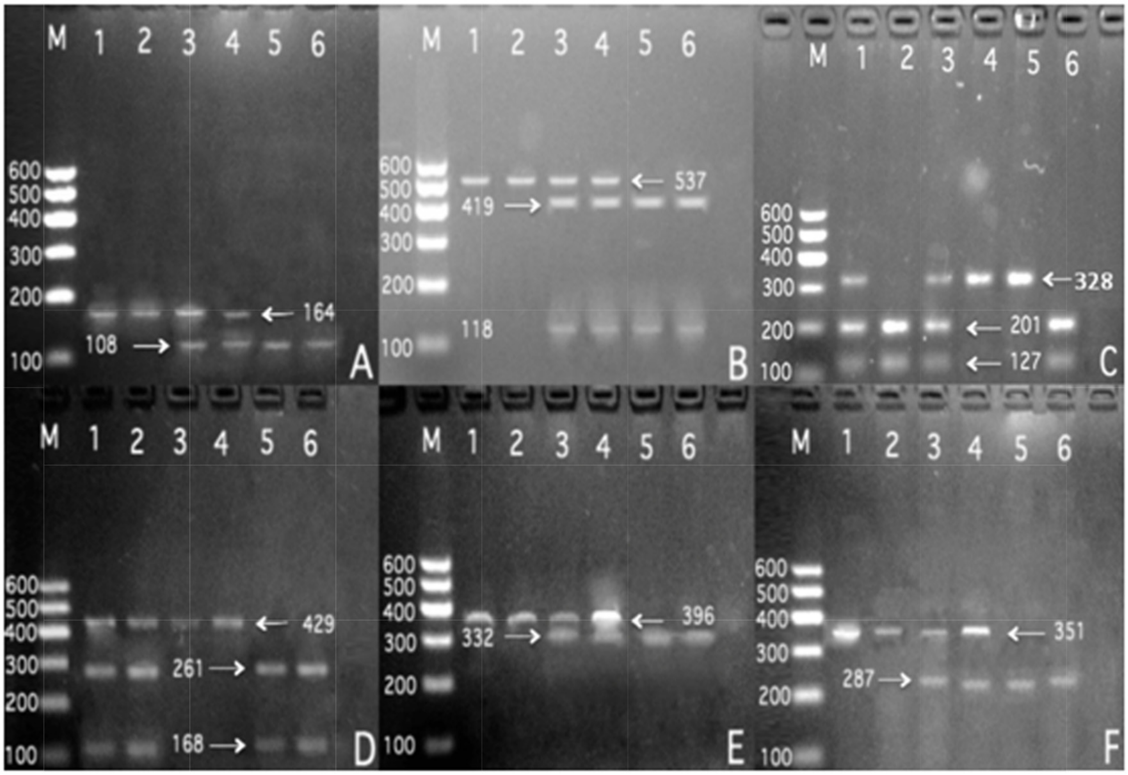

Supplementary Figure 2: Agarose gel electrophoresis (2%) of genotyping of the *PINX1* and *NAT2* SNPs. Lane M: DNA ladder 100 bp. The genotypes of 6 SNPs were as follow: **(A)** *NAT2* rs1961456: AA (lanes 1 and 2, 164 bp); GA (lanes 3 and 4, 164-, 108- and 56-bp); and GG genotype (lanes 5 and 6, 108- and 56-bp). **(B)** *NAT2* rs1495743: CC (lanes 1 and 2, 537 bp); GC (lanes 3 and 4, 328-, 201- and 127-bp); and GG genotype (lanes 5 and 6, 201- and 127-bp). **(C)** *NAT2* rs1799930: AA (lanes 4 and 5, 328 bp); GA (lanes 1 and 3, 328-, 201- and 127-bp); and GG genotype (lanes 2 and 6, 201- and 127-bp). **(D)** *NAT2* rs1799931: AA (lanes 3 and 4, 429 bp); GA (lanes 1 and 2, 429-, 261- and 168-bp); and GG genotype (lanes 5 and 6, 261- and 168-bp). **(E)** *PINX1* rs11776767: CC (lanes 1 and 2, 396 bp); GC (lanes 3 and 4, 396-, 332- and 74-bp); and GG (lanes 5 and 6, 332- and 74-bp). **(F)** *PINX1* rs6601530: AA (lanes 1 and 2, 351 bp); GA (lanes 3 and 4, 351-, 287- and 64-bp); and GG genotype (lanes 5 and 6, 287- and 64-bp). The less than 90-bp fragment was invisible in the gel owing to its fast migration speed.

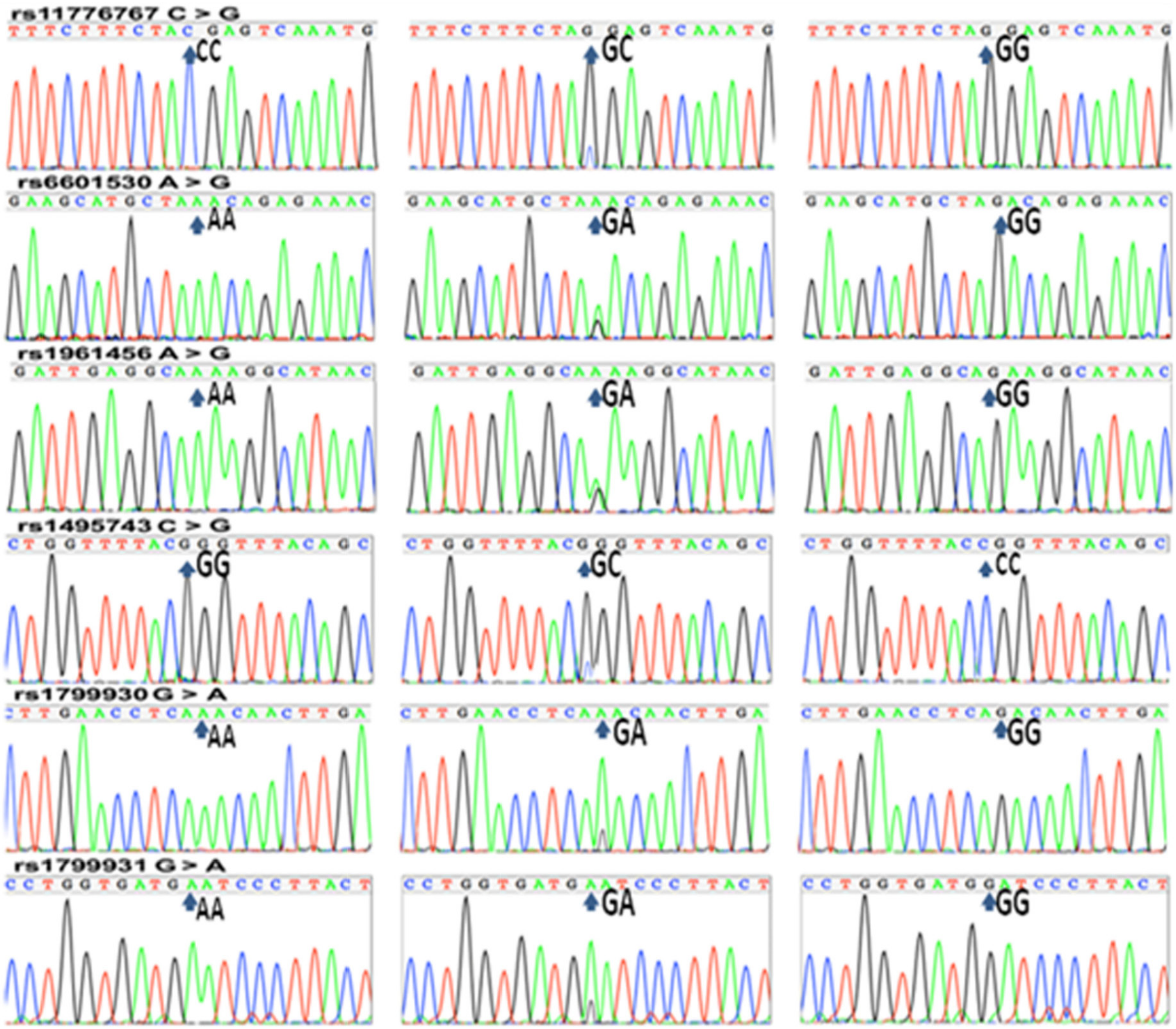

Supplementary Figure 3: The parts of the nucleotide direct sequencing results of the *PINX1* and *NAT2* SNPs. *PINX1,* PIN2/TERF1-interacting Telomerase Inhibitor 1; *NAT2,* N-acetyltransferase 2.

Supplementary Table 1: Characteristics of the *PINX1* and *NAT2* SNPs

SNP ID (rs#)	HGVS Name	Chr:Position	Contig	Contig Pos	SNP to Chr	MAF/Minor
PINX1						
rs11776767	NM_001284356.1: c.302-175C>G	8:10826419	NT_077531.5	3159292	Fwd	C=0.4353/2180 (1000 Genomes)
rs6601530	NM_001284356.1: c.394+12390A>G	8: 10813762	NT_077531.5	3146635	Fwd	G=0.4239/2123 (1000 Genomes)
NAT2						
rs1961456	NM_000015.2: c.-6-1799A>G	8: 18398199	NT_167187.2	6113854	Fwd	G=0.4115/2061 (1000 Genomes)
rs1495743	NC_000008.10: g.18273300G>C	8: 18415790	NT_167187.2	6131445	Fwd	C=0.3508/1757 (1000 Genomes)
rs1799930	NM_000015.2: c.590G>A	8:18400593	NT_167187.2	6116248	Fwd	A=0.2650/1327 (1000 Genomes)
rs1799931	NM_000015.2: c.857G>A	8: 18400860	NT_167187.2	6116515	Fwd	A=0.0773/387 (1000 Genomes)

Supplementary Table 2: The sequences of forward and backward primers of the *PINX1* and *NAT2* SNPs

SNP	Primer sequence	Annealing temperature	PCR product
PINX1			
rs11776767	GCAACTCAGGACAAACACGT	59°C	396 bp
	AAATTTCTGGAGGGCAGCCT		
rs6601530	TCCACCAAGCAAAGCAGAAG	59°C	351 bp
	ACTCCGAAACCAGAGTGAGG		
NAT2			
rs1961456	TGGAGTGTGTATGCTGGAGT	58°C	164 bp
	GACAGGTCTTGCTGGGTTTC		
rs1495743	ACCTTCTCACTGTGCCTTCA	58°C	537 bp
	GGTTGGGGCCTCTTTCTTCT		
rs1799930	CAGATGTGGCAGCCTCTAGA	58°C	328 bp
	GGATGAAGCCCACCAAACAG		
rs1799931	GGGTTTACTGTTTGGTGGGC	59°C	429 bp
	TCCCAAGATAATCACAGGCCA		
